# Coniferous-to-broadleaved forest conversion in subtropical coastal sandy lands enhances soil component respiration: coupled driving effects and mechanisms for improved carbon sequestration stability

**DOI:** 10.3389/fpls.2026.1825439

**Published:** 2026-06-03

**Authors:** Xin Huang, Wei Gao, Chao Gong, Xiaoxia Zeng, Xianzhen Zhou, Gongfu Ye

**Affiliations:** 1 College of Forestry, Fujian Agriculture and Forestry University, Fuzhou, Fujian, China; 2 Institute of Forest Ecology and Carbon Sink Measurement, Fujian Forestry Vocational and Technical College, Nanping, Fujian, China; 3 Fujian Academy of Forestry, Fuzhou, Fujian, China

**Keywords:** carbon sequestration stability, coastal protection forest, coupled driving, soil component respiration, subtropical coastal sandy land, temperature sensitivity (*Q*_10_), tree species conversion

## Abstract

**Introduction:**

Coniferous_to_broadleaved forest conversion reshapes soil carbon cycling in coastal sandy ecosystems, yet its regulation on component soil respiration and thermal sensitivity remains poorly quantified. To explore the regulatory effects and underlying mechanisms of such vegetation shift on soil carbon cycling in subtropical coastal sandy lands, we carried out this comparative field study.

**Methods:**

We investigated the conversion from *Pinus elliottii* (coniferous forest) to *Eucalyptus urophylla* × *E. grandis* (broadleaved forest) using a paired adjacent plot design and two_year continuous *in_situ* observations. Key indicators including soil respiration components, litter properties, fine root biomass, microbial activity and soil microclimate were monitored.

**Results:**

Results showed that the conversion significantly increased total soil respiration (by 25.52%), root respiration (by 62.74%), and heterotrophic respiration (by 9.01%). This promotion was driven by the coupled effects of improved litter quality (low C/N ratio and lignin content), a sharp increase in fine root biomass (by 272.5%), and enhanced microbial activity. It also notably reduced the temperature sensitivity (*Q*_10_ from 2.29 to 1.55) of soil respiration, with root respiration becoming nearly temperature_insensitive (*Q*_10_=1.25). Additionally, the explanatory power of soil temperature for respiration decreased significantly (from 76% to 37.7%), while the regulatory role of litter quality, fine root biomass, and soil microbial activity became prominent, and soil moisture did not act as a limiting factor for soil respiration throughout the study period.

**Discussion:**

This conversion achieves a virtuous cycle of “high carbon turnover and high carbon sequestration”, clarifying the above_belowground coupling mechanism of soil carbon dynamics. Our findings thereby provide important scientific support for the optimization of coastal protection forests and the enhancement of carbon sequestration capacity in fragile coastal ecosystems.

## 1 Introduction

As the second-largest carbon flux process in terrestrial ecosystems following photosynthesis, soil respiration releases 68–100 Pg C annually (Bond-Lamberty and Thomson, 2010), approximately tenfold the global carbon emissions from fossil fuels (Schlesinger and Andrews, 2000), and plays an irreplaceable and pivotal role in regulating the global carbon cycle and climate change (Valentini et al., 2000). This process is composed of two major components: autotrophic respiration (derived from the metabolism of plant roots and rhizosphere organisms) and heterotrophic respiration (resulting from the decomposition of organic matter by microorganisms) (Kotroczo et al., 2023), which differ significantly in their bioecological mechanisms and environmental response characteristics (Yu et al., 2015). Within forest ecosystems, the soil carbon pool stores approximately 73% of total ecosystem carbon (Lal, 2010); as the core pathway of carbon output, soil respiration contributes 60%–90% of the total forest carbon flux (Schimel et al., 2001) and exerts a decisive effect on maintaining the global carbon balance (Kudeyarov, 2023).

Alterations in forest type and tree species composition can directly reshape the component structure and flux pattern of soil respiration, which constitutes a key link driving the dynamic evolution of the ecosystem carbon cycle (Yang et al., 2004b; Hu et al., 2018). Previous studies have demonstrated that tree species conversion during afforestation significantly influences the spatiotemporal variability of soil respiration by regulating key ecological processes, including litter input characteristics, fine root growth dynamics, and soil microbial community structure (Guo et al., 2016; Liu et al., 2019). However, most existing studies are confined to static comparisons, making it challenging to distinguish whether observed differences stem from stand structure itself or changes in ecological processes induced by tree species replacement. Empirical evidence also exhibits notable discrepancies: soil respiration is significantly reduced following the conversion of subtropical evergreen broadleaved forests to coniferous plantations (Liu et al., 2019); conversion of natural forests to coniferous plantations may increase the proportion of heterotrophic respiration (Zhao et al., 2022); whereas coniferous-to-broadleaved forest conversion typically promotes enhanced soil respiration due to optimized characteristics such as reduced litter carbon-to-nitrogen (C/N) ratio and increased fine root biomass (Huang et al., 2014). This inconsistency in conclusions further underscores the necessity and urgency of elucidating the driving mechanisms of tree species conversion within specific habitat contexts.

As a typical fragile ecosystem under the interaction of land and sea, coastal sandy lands are generally characterized by poor soil quality, weak water-holding capacity, significant salt stress, and frequent typhoon disturbances (Hu et al., 2022; Jin et al., 2022). The construction of ecological protection barriers in these regions is critical for the sustainable development of coastal zones. Although existing studies have revealed the following: natural secondary forests dominated by zonal evergreen broadleaved trees generally exhibit superior soil carbon and nitrogen contents as well as microbial indicators compared to plantations; stand age, litter input, and root characteristics are key factors regulating soil carbon and nitrogen storage (Gao et al., 2020); Although previous studies have revealed that coniferous-to-broadleaved conversion strongly affects soil respiration, most investigations have focused on broad functional groups rather than specific species pairs such as (*Pinus elliottii* vs. *Eucalyptus urophylla × E. grandis*) *(*Tiwari and Meena, 2025). Case studies have demonstrated that replacing Pinus sylvestris with Quercus ilex reduced soil respiration by 36% in Mediterranean forests (Barba et al., 2016), whereas conversion from pine to broadleaved species significantly increased soil CO_2_ emission by improving litter quality and fine root biomass in subtropical forests (Huang et al., 2014; Lee et al., 2023). In coastal sandy lands, tree species identity strongly regulates soil respiration, microbial biomass, and temperature sensitivity (*Q*_10_) (Gao et al., 2017), yet comparative studies between coniferous and broadleaved plantations remain extremely limited. More importantly, no empirical studies have clarified the divergent driving mechanisms of soil carbon cycling between coastal sandy habitats and inland forests under analogous tree species conversion. These critical knowledge gaps restrict the extension of inland-derived carbon cycle theories to coastal protection forest ecosystems, and highlight the urgent necessity of species-specific research in coastal sandy regions.

Nevertheless, in the context of frequent afforestation and reforestation practices in coastal sandy lands, several key scientific questions regarding the mechanisms underlying the impact of coniferous-to-broadleaved forest conversion on soil carbon storage capacity remain unanswered: How does tree species conversion alter soil respiration and its components? What are the core driving factors? How will these changes affect the carbon sequestration capacity of ecosystems and the ecological benefits of protection forests? Most importantly, as a core functional trait regulating carbon decomposition and sequestration, whether the temperature response mechanism of microbial carbon use efficiency (CUE) is involved in the regulation of soil respiration under tree species conversion has not been clearly verified in the unique habitat of coastal sandy lands (Yu et al., 2025).

In light of this, the present study focuses on coastal sandy lands along the southeast coast of China, taking the typical afforestation model of converting *Pinus elliottii* forests (coniferous forests) to *Eucalyptus urophylla × E. grandis* forests (broadleaved forests) as the research object. The “block replacement” model formed by these two tree species during long-term afforestation practices has emerged as an important approach for optimizing stand structure and improving protective functions. Based on the comparable annual litter production of the two stands, and the distinct characteristics of Eucalyptus urophylla × E. grandis litter (lower C/N ratio and higher fine root biomass), the core hypothesis of this study is proposed as follows: Conversion from *Pinus elliottii* coniferous forests to *Eucalyptus urophylla × E. grandis* broadleaved forests in coastal sandy lands will significantly increase soil respiration rate. This change is primarily driven by the coupled effects—defined here as the synergistic interaction and combined regulation of aboveground litter quality, belowground fine root biomass, and soil microbial activity—rather than by any single factor alone.

Centering on the aforementioned hypothesis, this study aims to achieve the following research objectives: (1) Quantify the differences in total soil respiration and its component fluxes (root respiration, heterotrophic respiration) and their seasonal dynamics between the two converted forest types. (2) Clarify the response of soil respiration to soil temperature and moisture, and quantify changes in its temperature sensitivity (*Q*_10_) after conversion.; (3) Uncover the coupled driving mechanisms of litter quality, fine root biomass, and soil microbial activity on soil respiration, and reveal their regulatory effects on carbon sequestration stability in coastal sandy lands. The findings of this study are expected to enhance the theoretical understanding of carbon processes in fragile ecosystems and provide scientific basis and practical support for the optimization of tree species configuration in coastal protection forests, as well as the improvement of ecosystem carbon sequestration, sink enhancement, and comprehensive protective benefits.

## 2 Materials and methods

### 2.1 Study site and experimental design

**Figure 1:**
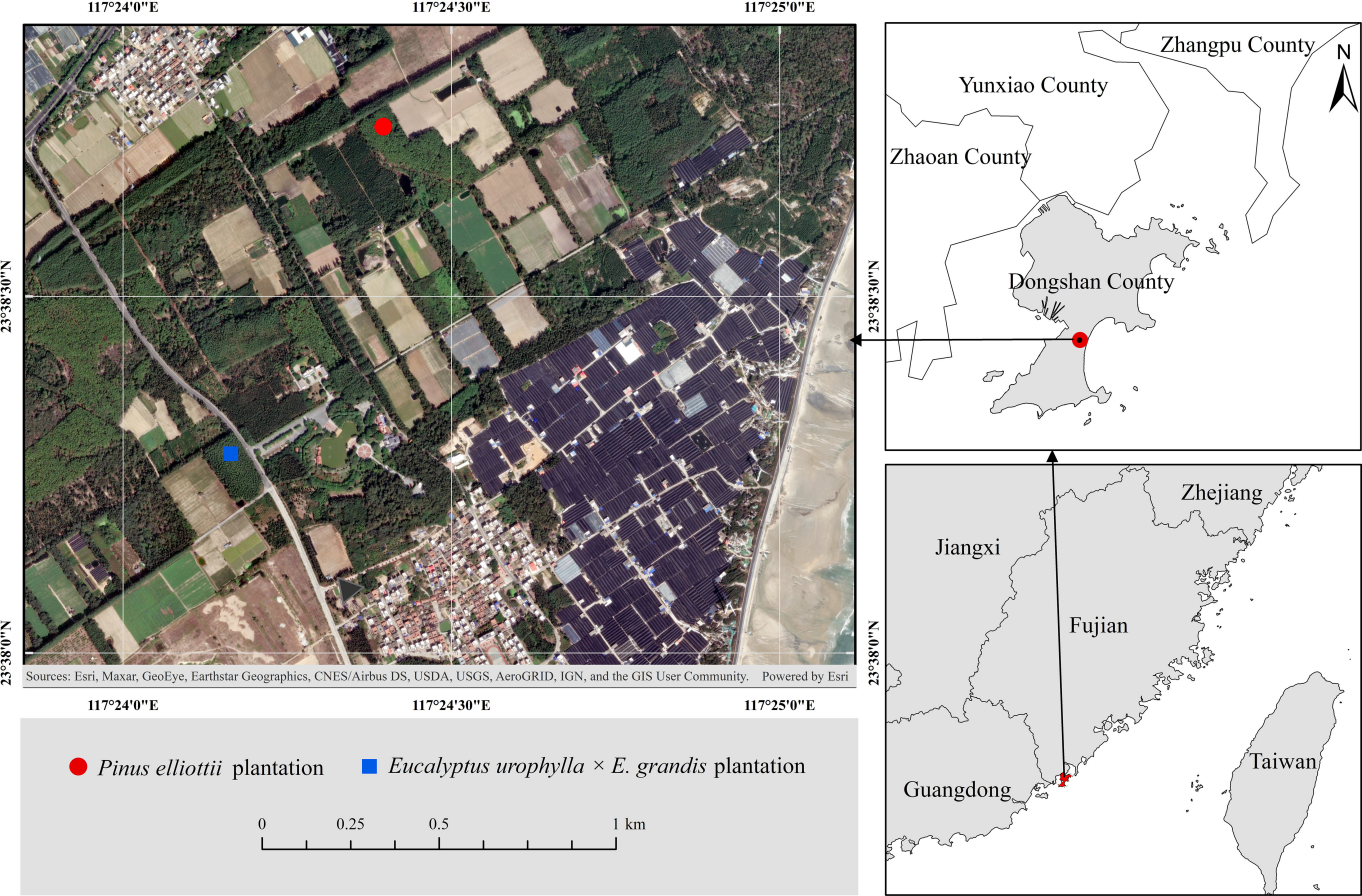
Study area overview map.

The study was conducted at Chishan State-owned Protection Forest Farm in Dongshan County, Fujian Province, located in southeastern China (23°38′ N, 117°24′ E), which belongs to a subtropical marine monsoon climate. The spatial location and distribution of the study site are clearly illustrated in **Figure 1**. The specific geographic coordinates of the *Pinus elliottii* plantation plot are 117°24′23.8932″ E, 23°38′44.1528″ N, and those of the *Eucalyptus urophylla × E. grandis* plantation plot are 117°24′09.8532″ E, 23°38′16.2024″ N.

**Figure 2:**
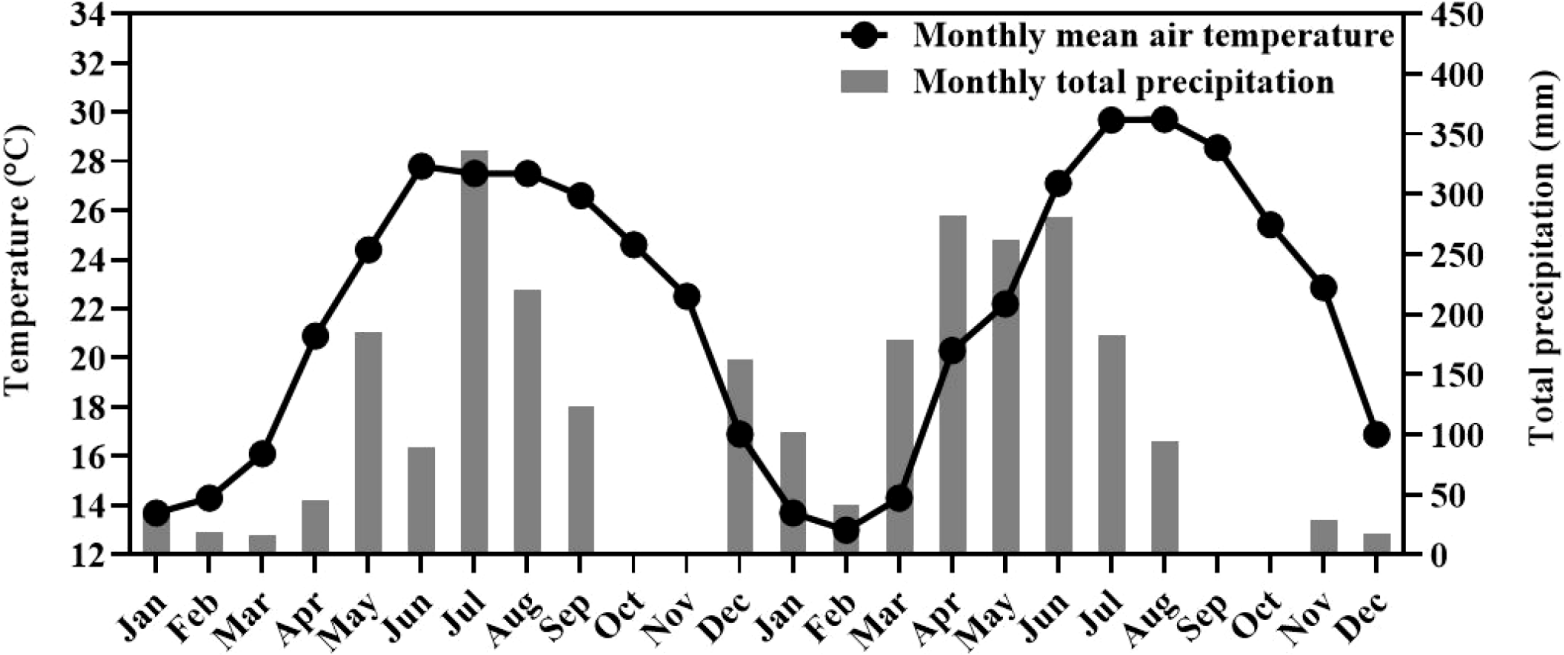
Monthly mean air temperature and precipitation in the study area from January 2021 to December 2022 (n=1).

During the study period from 2021 to 2022, the average annual temperature was 21.9 °C, and the annual precipitations in 2021 and 2022 were 1235 mm and 1474 mm, respectively, with 77.4% of the precipitation concentrated between May and September; the precipitation in the dry season (January–April) of 2022 was significantly higher than that in the same period of 2021 (**Figure 2**).

The study plots were derived from *Pinus elliottii* plantations established in 1976. Due to long-term disturbances such as typhoons, pests, and diseases, a large area of trees died and lodged. In April 2004, regeneration afforestation was carried out on this stand: part of the area continued to be planted with *Pinus elliottii* plantations, and the other part was replaced with *Eucalyptus urophylla × E. grandis* plantations, forming a block mixed pattern. Both stand types had coastal sandy soil with low nutrient content and a thickness of 80–100 cm. The main stand characteristics and topsoil (0–10 cm) physical and chemical properties of *Pinus elliottii* and *Eucalyptus urophylla × E. grandis* plantations are shown in **Table 1**.

**Table 1:** Main stand characteristics and physicochemical properties of topsoil (0–10 cm) in *Pinus elliottii* and *Eucalyptus urophylla × E. grandis* plantations (n=4).

Parameters	*Pinus elliottii*	*Eucalyptus urophylla × E. grandis*
Elevation (m)	18.77	18.71
Stand Age (a)	16	16
Average DBH (cm)	21	15.6
Average Tree Height (m)	13.9	11.4
Stand Density (plant·hm^-^²)	1487	1290
Canopy Density	0.8	0.8
Annual Litterfall (t·hm^-^²·a^-1^)	12.99 ± 1.48a	12.32 ± 1.57a
Litter C Content (g·kg^-^¹)	499.72 ± 4.84a	473.42 ± 28.36a
Litter N Content (g·kg^-^¹)	6.60 ± 6.58a	9.20 ± 9.21b
Litter C: N	77.12 ± 10.59a	51.55 ± 4.36a
Litter Lignin Content (g·kg^-^¹)	365.9 ± 0.01a	179.92 ± 7.54b
Litter Lignin: N	56.50 ± 8.02a	19.61 ± 1.71b
Fine Root Biomass (t·hm^-^²)	0.80 ± 0.84a	2.98 ± 1.29b
Soil pH	4.65 ± 0.13a	5.01 ± 0.07b
Soil Bulk Density (g·cm^-^³)	1.24 ± 0.02a	1.35 ± 0.01a
Soil C Content (g·kg^-^¹)	5.70 ± 2.65a	6.65 ± 2.67a
Soil N Content (g·kg^-^¹)	4.21 ± 0.95a	0.54 ± 0.92a
Soil C:N	3.68 ± 4.77a	11.96 ± 2.90a
Clay (< 0.002 mm)	<1%a	<1%a
Silt (0.002–0.05 mm)	<1%a	<1%a
Sand (> 0.05 mm)	>98%a	>98%a
Available Phosphorus (mg·kg^-^¹)	0.61 ± 0.11a	0.57 ± 0.31a
Exchangeable Magnesium (mg·kg^-^¹)	0.33 ± 0.08a	0.28 ± 0.05a
MBC (mg·kg^-^¹)	42.11 ± 20.50a	118.69 ± 94.63a
DOC (mg·kg^-^¹)	40.39 ± 11.58a	68.13 ± 28.00a

The values in the table are mean ± standard error, and different letters indicate significant differences (*p* < 0.05).

In June 2020, four permanent plots (20 m × 20 m each, totaling 8 plots) were established in each of the two stand types, with a spacing of at least 10 m between plots. Three subplots (2 m × 2 m each, 3 m apart) were randomly set in each plot, and randomly assigned to three treatments: (1) Litter removal: All surface litter in the subplot was removed. To intercept and prevent litter from falling into the subplot, a 2.5 m × 2.5 m nylon net (mesh size 1 mm × 1 mm) was erected 0.5 m above the ground, and litter and impurities attached to the nylon net were cleaned every two weeks. (2) Root exclusion: The trench digging method was used to distinguish soil respiration components. A trench 30 cm wide and 70 cm deep was dug around the subplot to ensure no residual roots remained under the trench. After embedding a double-layer 0.1 mm nylon net in the trench, the excavated soil was carefully backfilled and compacted, and then all vegetation on the ground was carefully cut off to minimize soil disturbance, and no live plants were allowed to grow in the trench plot throughout the study period. (3) Control treatment: The natural state was maintained, and no treatment was applied to the subplot.

### 2.2 Determination of soil respiration and microclimate

A PVC soil respiration collar (inner diameter 20 cm, height 7 cm) was installed at the center of each subplot, inserted to a depth of 4 cm for soil respiration measurement. Six months after the root exclusion treatment, from January 2021 to December 2022, the Li-8100 Automated Soil CO_2_ Flux System (Li-Cor Inc., Lincoln, NE, USA) was used to measure the soil respiration rate of subplots under different treatments. Monthly measurements were conducted on sunny days between 09:00 and 11:00 (Beijing Time), as data collected during this period can effectively represent the daily average value (Xu and Qi, 2001). Before measurement, plants on the ground within the respiration collar were carefully cut off to avoid interference. Meanwhile, a handheld long-pole thermometer (SK-250WP, Sato Keiryoki Mfg. Co. Ltd, Tokyo, Japan) was used to determine soil temperature, and a time-domain reflectometer (TDR300, Spectrum Technologies Inc., Plainfield, IL, USA) was used to measure the volumetric water content of the 0–10 cm soil layer. For each collar, four consecutive repeated measurements were taken, and the average value was used as the final observation to minimize random measurement error. Meteorological data, including monthly average temperature and precipitation, were provided by an automatic weather station within 5 km of the experimental site. The formulas for calculating soil respiration components were as follows: (1) Root respiration rate = Total soil respiration rate - Soil respiration rate in root-exclusion subplots; (2) Heterotrophic respiration rate = Soil respiration rate in root-exclusion subplots; (3) Litter layer respiration rate = Total soil respiration rate - Soil respiration rate in litter-removal subplots.

### 2.3 Litter and fine root production

Five litter collection frames (1 m × 1 m in size, nylon mesh with 1 mm mesh) were randomly placed 0.3 m below the canopy of each plot. Litter was collected monthly from March 2021 to February 2022 to estimate the annual litter production. The collected litter was dried to a constant weight at 60 °C and then weighed. The dried litter was ground and sieved (100 mesh), and the carbon and nitrogen concentrations of the ground litter were determined using an Elementar Vario EL III CN analyzer. The lignin content was measured according to the method described by Kirk et al (Kirk and Obst, 1988). In July 2021, 10 soil cores (1.5 m deep, 5 cm in diameter) were collected along the diagonal of each plot. The soil cores were soaked in deionized water for 30 min and then sieved through a 0.5 mm sieve to separate fine roots (diameter < 2 mm) from the soil matrix. Live fine roots and dead fine roots were distinguished by visual observation of root color and morphological characteristics: live fine roots were identified by their fresh creamy white, pale yellow or light brown stele (xylem) with a smooth and intact epidermis, while dead fine roots were dark brown or black in color, with a shrunken, brittle epidermis and discolored stele. After separation, live fine roots were rinsed with deionized water to remove residual soil particles, and their biomass was estimated using the method proposed by Yang et al. (2004a).

### 2.4 Determination of soil physicochemical properties and microbial community

In December 2021, 10 sampling points were evenly selected along the diagonal of each plot, and topsoil (0–10 cm) was collected using a soil auger with an inner diameter of 5 cm. A 500 g sample was collected from each plot and evenly divided into two parts, which were quickly brought back to the laboratory in an ice box. One part was sieved through a 2 mm sieve and stored at -20 °C for soil microbial community analysis as soon as possible. The other part of the air-dried soil sample was ground and sieved through a 0.149 mm sieve to determine total soil carbon and total nitrogen. In addition, fresh topsoil (0–10 cm) samples were collected from control and root-exclusion subplots in different plots in June 2021, September 2021, January 2022, and May 2022 to determine soil dissolved organic carbon (DOC) and microbial biomass carbon (MBC). The specific methods were as follows: fresh soil samples from each treatment were sieved through a 2 mm sieve, immediately extracted with deionized water and filtered, and the DOC content in the filtrate was determined using a TOC-VCPH/CPN+ analyzer (Shimadzu, Japan); MBC was analyzed by the chloroform fumigation-extraction method (Brookes et al., 1985), the organic carbon content in the extract was determined using a TOC analyzer, and the MBC value was calculated with a conversion factor of 0.45 (Wu et al., 1990; Joergensen, 1996). The contributions of roots to soil DOC and MBC in the two stand types were calculated. Total soil nitrogen was determined using an elemental analyzer (Vario EL III, Elementar, Germany). Soil pH was measured with a pH meter after extraction at a soil-to-water ratio of 2.5:1. Available phosphorus was determined by the molybdenum-antimony anti-colorimetric method, and exchangeable magnesium was determined by atomic absorption spectrometry (Bao, 2000). The bulk density of topsoil was measured using the ring knife method.

The soil microbial community was analyzed using the phospholipid fatty acid (PLFA) method. The extraction of PLFAs referred to the method described by White et al. (1979). After separation by a silica gel column, PLFAs were identified according to their retention time in a gas chromatograph (Agilent 6890N, USA) combined with the MIDI microbial identification system (MIDI Inc., Newark, DE). Based on previous research results, the PLFA markers indicating specific microorganisms are shown in **Table 2**.

**Table 2:** Biomarkers of microbial types.

Microbial groups	PLFA markers
Gram-positive Bacteria (G^+^)	i14:0, i15:0, a15:0, i16:0, i17:0, a17:0
Gram-negative Bacteria (G^-^)	16:1 w9c, 16:1 w7c, cy 17:0, 18:1 w7c, 18:1 w5c, cy 19:0
Fungi	18:2 w6c, 18:1 w9c
Actinobacteria (Actino)	10 me 16:0, 10 me 17:0, 10 me 18:0
Arbuscular Mycorrhizal Fungi (AMF)	16:1 w5c

### 2.5 Data analysis

When analyzing soil respiration and its components, as well as environmental and plant parameters of each stand type, each plot was treated as an experimental unit, and the data from repeated measurements were averaged by plot before analysis. Independent samples t-test was used to analyze the differences in litter characteristics, soil physicochemical properties, and annual soil respiration fluxes between the two stand types. One-way analysis of variance (ANOVA) was employed to test the differences in soil respiration components, soil temperature, and soil moisture between the two stand types. Line charts and bar charts were plotted using GraphPad Prism 9.5.0 software, and correlation heatmaps between different soil respiration components and environmental factors were drawn using Origin 2021. Structural equation model (SEM) analysis was performed using the lavaan package in R Studio 4.5.2 software.

Linear and nonlinear regression models were used to describe the relationships between the seasonal variations of different soil respiration components and soil temperature and soil moisture, as seen in Equations 1–3. The temperature sensitivity (*Q*_10_) was calculated by Equation 4, and the annual fluxes of each soil respiration component were calculated by Equation 5. The specific formulas are as follows:

(1) $$R={\text{ae}}^{bT}$$

(2) R=aW+b

(3) $$R={\text{ae}}^{bT}W^{\text{c}}$$

(4) $$Q_{10}={\text{e}}^{10\text{b}}$$

(5) $$F=\frac{\bar{R}\times 12\times 3600\times 24\times D}{10^{6}}$$

Where: *R* is the soil respiration rate; *T* is the soil temperature at 10 cm depth (°C); *W* is the soil volumetric water content at 0–10 cm depth (% vol.); a, b and c are undetermined parameters; b in Formula (4) is the temperature coefficient in Formula (1); $\bar{R}$ is the monthly average soil respiration rate (μmol m^-^²·s^-^¹); D is the number of days in the month; *F* is the monthly soil respiration flux (g C·m^-^²); and the annual flux of each soil respiration component is the sum of monthly fluxes.

## 3 Results

### 3.1 Seasonal variations of total soil respiration and component respiration in two stand types

**Figure 3:**
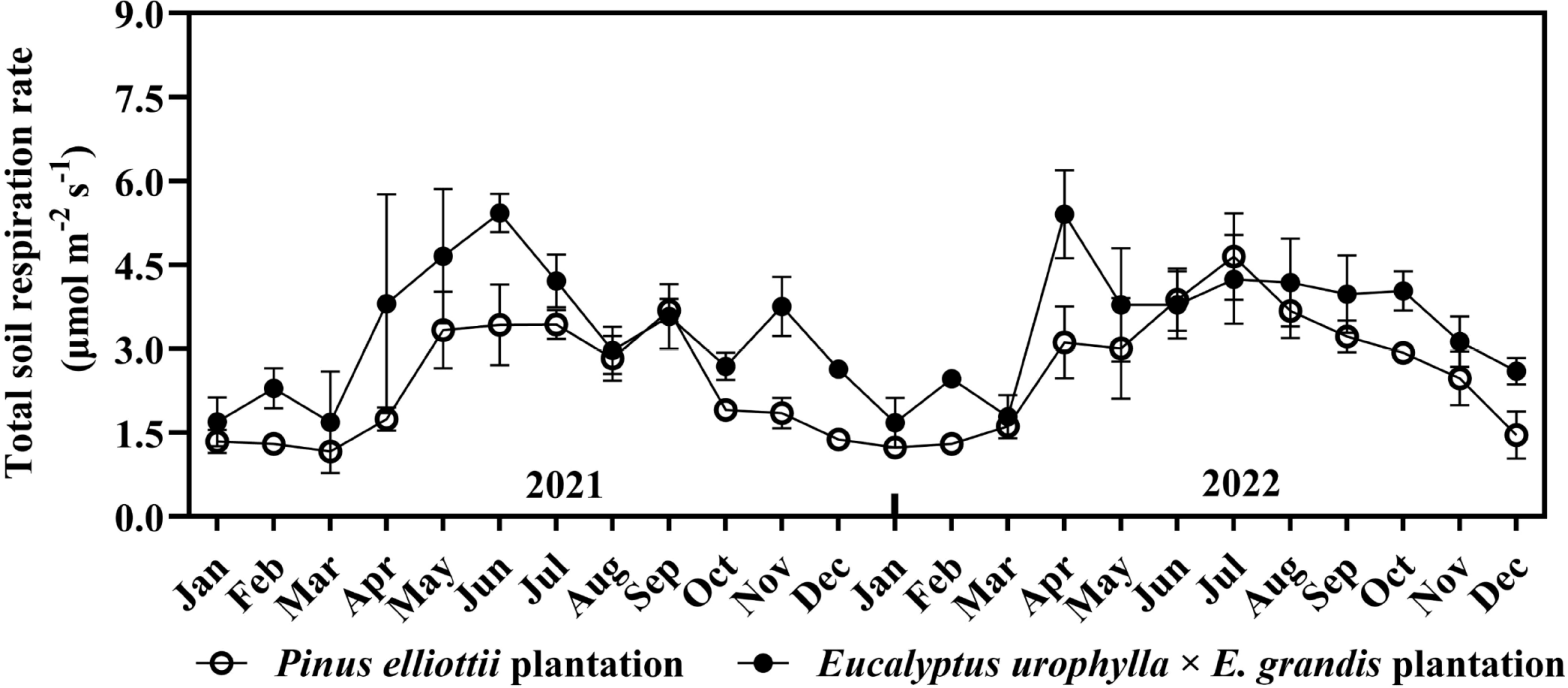
Monthly dynamics of total soil respiration rate in *Pinus elliottii* and *Eucalyptus urophylla × E. grandis* plantations (2021.1~2022.12). Error bars represent standard errors; same below (n=4).

From January 2021 to December 2022, total soil respiration in both *Eucalyptus urophylla × E. grandis* and *Pinus elliottii* plantations exhibited significant seasonal variations (**Figure 3**). Specifically, the peak value of total soil respiration rate in *Eucalyptus urophylla × E. grandis* plantations was observed in June 2021, reaching 5.43 μmol m^-^²·s^-^¹, while the valley value occurred in January 2022, at 1.68 μmol m^-^²·s^-^¹. In contrast, the peak total soil respiration rate in *Pinus elliottii* plantations appeared in July 2022, with the valley value recorded in January 2021. Analysis of variance (ANOVA) indicated that during the monitoring period, the average soil respiration rate of *Eucalyptus urophylla × E. grandis* plantations (3.35 μmol m^-^²·s^-^¹) was significantly higher than that of *Pinus elliottii* plantations (2.50 μmol m^-^²·s^-^¹).

**Figure 4:**
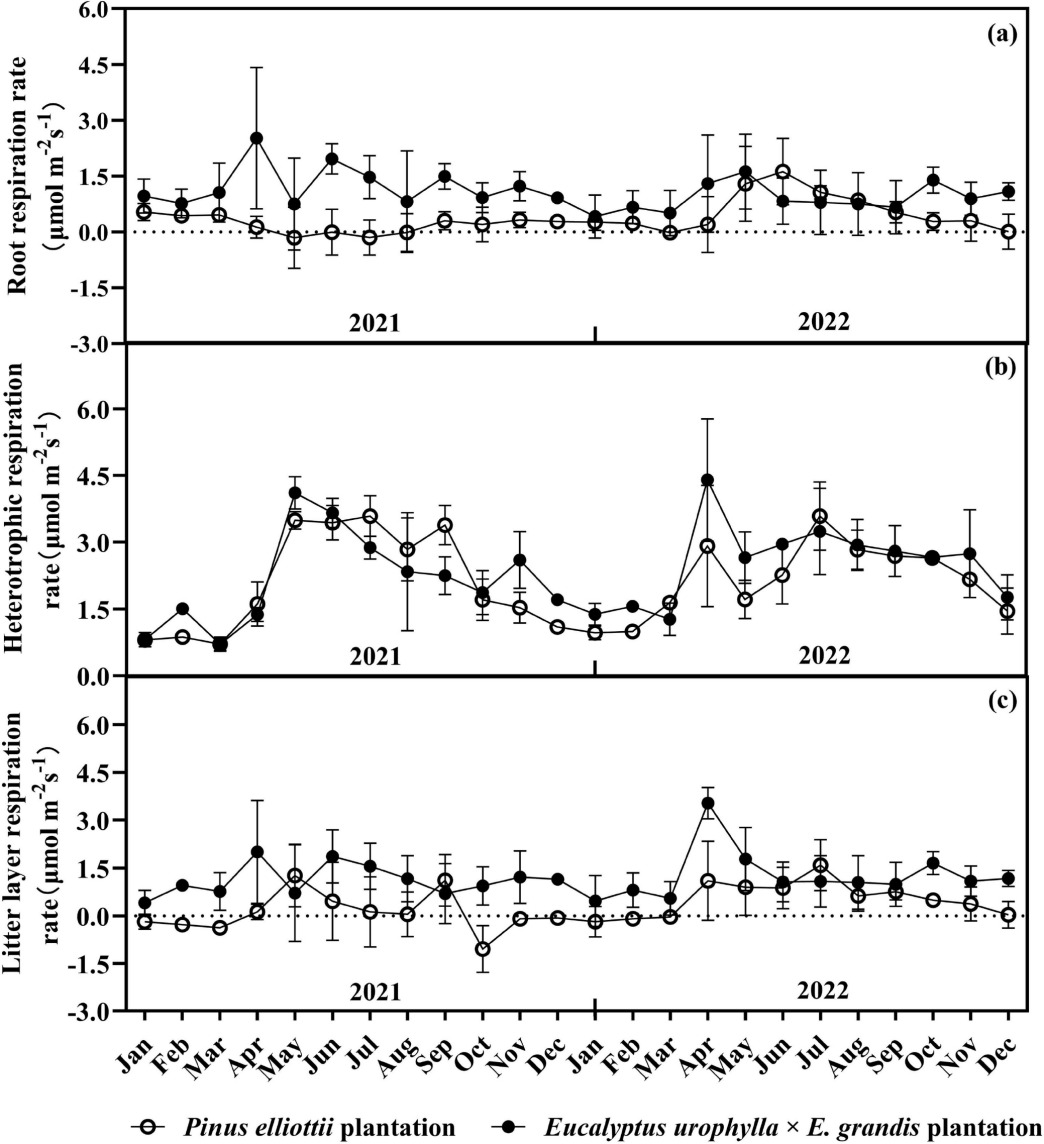
Monthly dynamics of root respiration rate **(a)**, heterotrophic respiration rate **(b)** and litter layer respiration rate **(c)** in *Pinus elliottii* and *Eucalyptus urophylla × E. grandis* plantations (2021.1~2022.12) (n=4).

From January 2021 to December 2022, root respiration (**Figure 4A**), heterotrophic respiration (**Figure 4B**), and litter layer respiration (**Figure 4C**) in both *Pinus elliottii* and *Eucalyptus urophylla × E. grandis* plantations also exhibited significant seasonal variations. Among them, the average root respiration rates of *Pinus elliottii* and *Eucalyptus urophylla ×E. grandis* plantations were 0.38 and 1.02 μmol m^-^²·s^-^¹, respectively; the average heterotrophic respiration rates were2.12and 2.33 μmol m^-^²·s^-^¹, respectively; and the average soil respiration rates in the litter layer were 0.31 and 1.19 μmol m^-^²·s^-^¹, respectively. ANOVA results indicated that the average root respiration, heterotrophic respiration, and litter layer respiration rates in *Eucalyptus urophylla × E. grandis* plantations were all significantly higher than those in *Pinus elliottii* plantations (*p* < 0.05).

### 3.2 Annual fluxes of soil respiration in two stand types

As presented in **Table 3**, the conversion from *Pinus elliottii* to *Eucalyptus urophylla × E. grandis* plantations elevated the annual fluxes of all soil respiration components. Specifically, the annual total soil respiration flux rose from 946 g C·m^-^²·a^-^¹ to 1267 g C·m^-^²·a^-^¹, a rise of 25.33%; the annual root respiration flux climbed from 143 g C·m^-^²·a^-^¹ to 386 g C·m^-^²·a^-^¹, with a relative gain of 62.95%; the annual heterotrophic respiration flux advanced from 802 g C·m^-^²·a^-^¹ to 881 g C·m^-^²·a^-^¹, corresponding to an increment of 8.96%; and the litter layer respiration flux soared from 117 g C·m^-^²·a^-^¹ to 451 g C·m^-^²·a^-^¹.

**Table 3:** Changes in annual soil respiration flux after conversion of *Pinus elliottii* to *Eucalyptus urophylla × E. grandis* plantations (n=4).

Soil respiration component	Annual flux (g C·m^-^²·a^-^¹)	
	*Pinus elliottii*	*Eucalyptus urophylla × E. grandis*
Total soil respiration	946b	1267a
Root respiration	143b	386a
Heterotrophic respiration	802a	881a
Litter layer respiration	117b	451a

Values in the same row followed by different lowercase letters indicate significant differences between the two forest types at the 0.05 level according to the LSD, least significant difference test.

### 3.3 Soil temperature, moisture and litter production in two stand types

**Figure 5:**
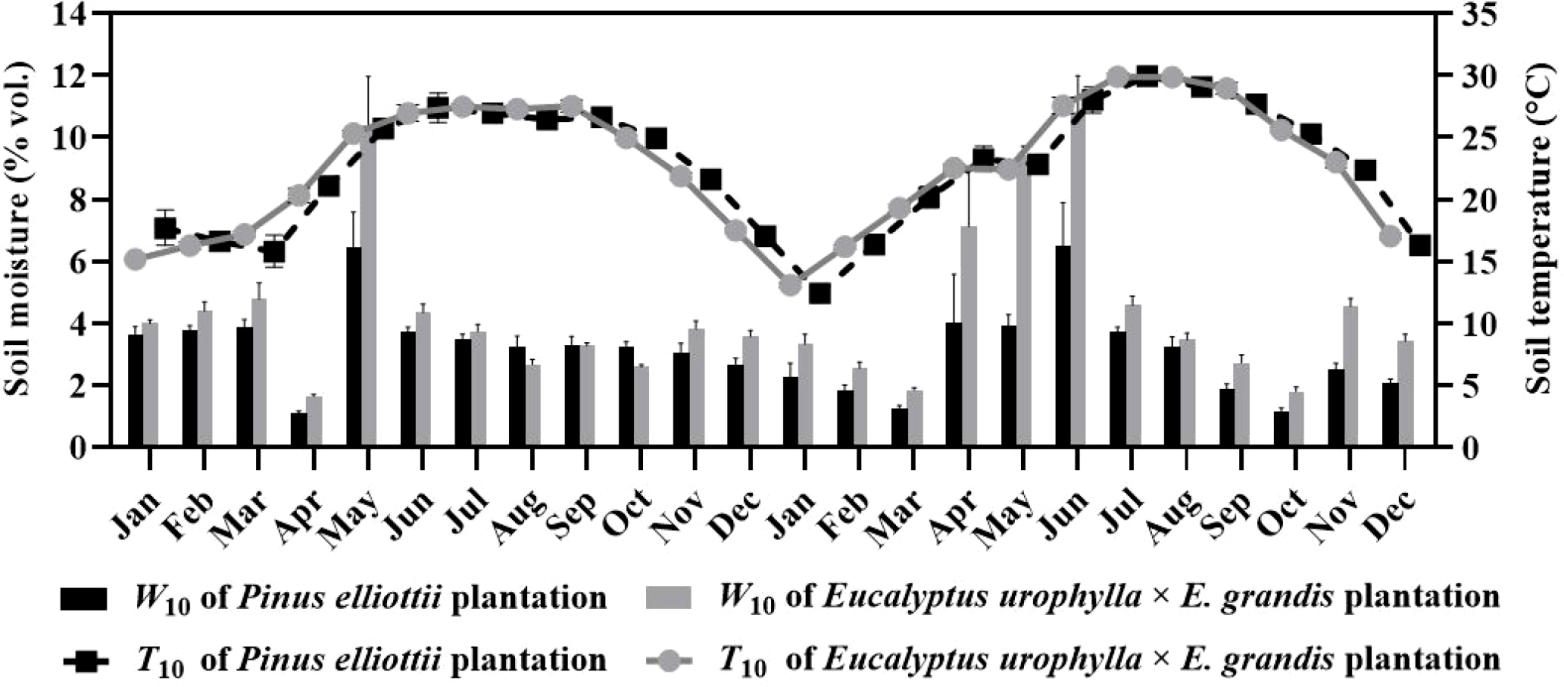
Monthly variations in soil temperature (*T*_10_) and soil volumetric water content (*W*_10_, % vol.) at 10 cm depth in *Pinus elliottii* and *Eucalyptus urophylla × E. grandis* plantations (n=4).

As shown in **Figure 5**, from January 2021 to December 2022, the overall temporal patterns of soil temperature and moisture at 10 cm depth were consistent between the two stand types. Soil temperature (*T*_10_) ranged from 13.07 °C to 29.87 °C, with no significant difference between the two stands across months (*p* = 0.68). Soil moisture (*W*_10_) varied from 1.01% to 11.8%, and the mean soil moisture in *Pinus elliottii* plantations was significantly lower than that in *Eucalyptus urophylla × E. grandis* plantations (*p* = 0.01).

**Figure 6:**
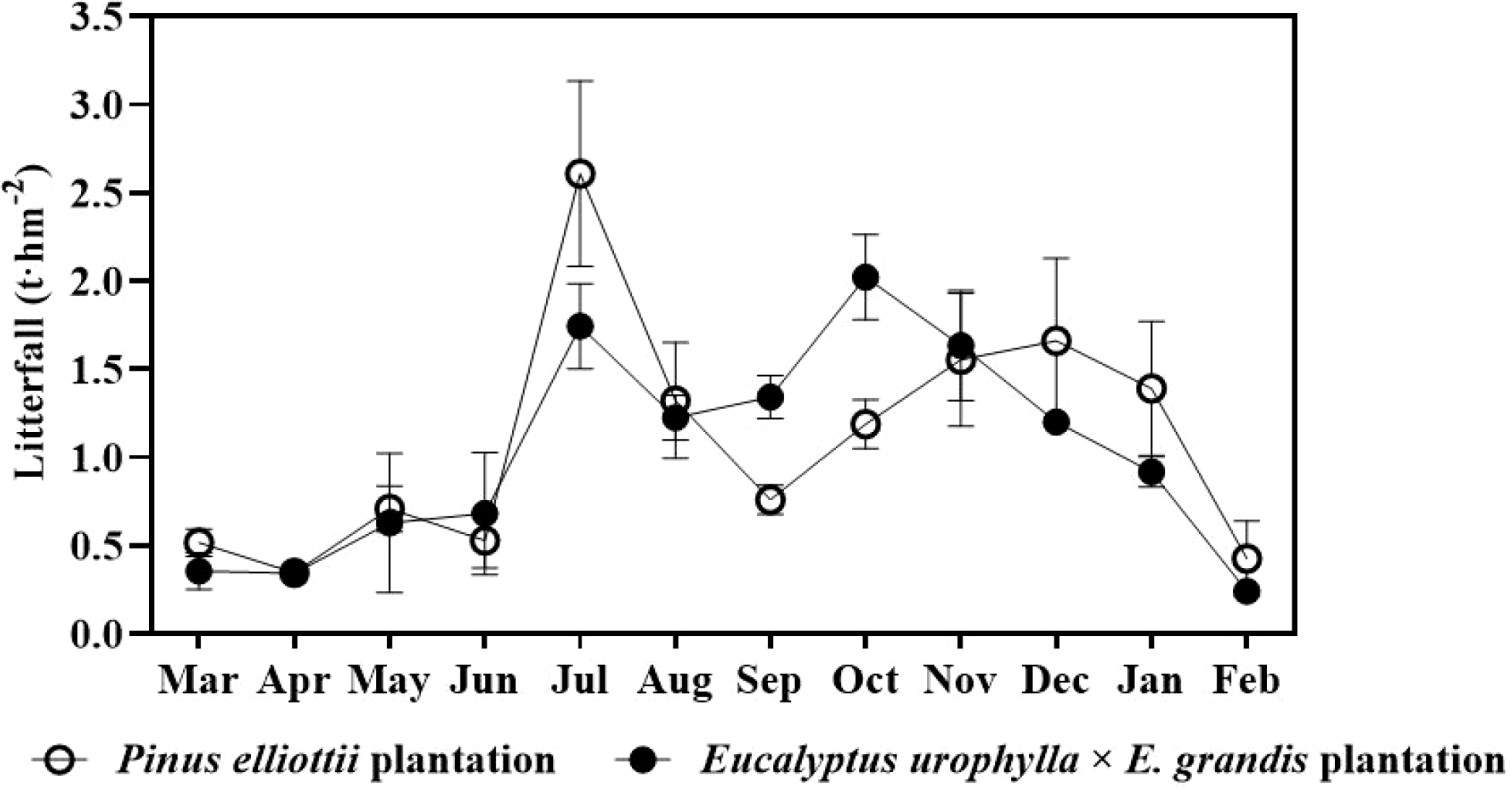
Monthly variation in litterfall of *Pinus elliottii* and *Eucalyptus urophylla × E. grandis* plantations (2021.3~2022.2) (n=4).

Litter production exhibited distinct monthly variations in both *Pinus elliottii* and *Eucalyptus urophylla × E. grandis* plantations (**Figure 6**). Litterfall in *Pinus elliottii* plantations peaked in July 2021, whereas that in *Eucalyptus urophylla × E. grandis* plantations peaked in October. Annual total litterfall was 12.99 t·hm^-^² in *Pinus elliottii* plantations, slightly higher than the 12.32 t·hm^-^² in *Eucalyptus urophylla × E. grandis* plantations, with no significant difference between the two stands (*p* = 0.600).

### 3.4 Soil microbial community structure in two stand types

**Figure 7:**
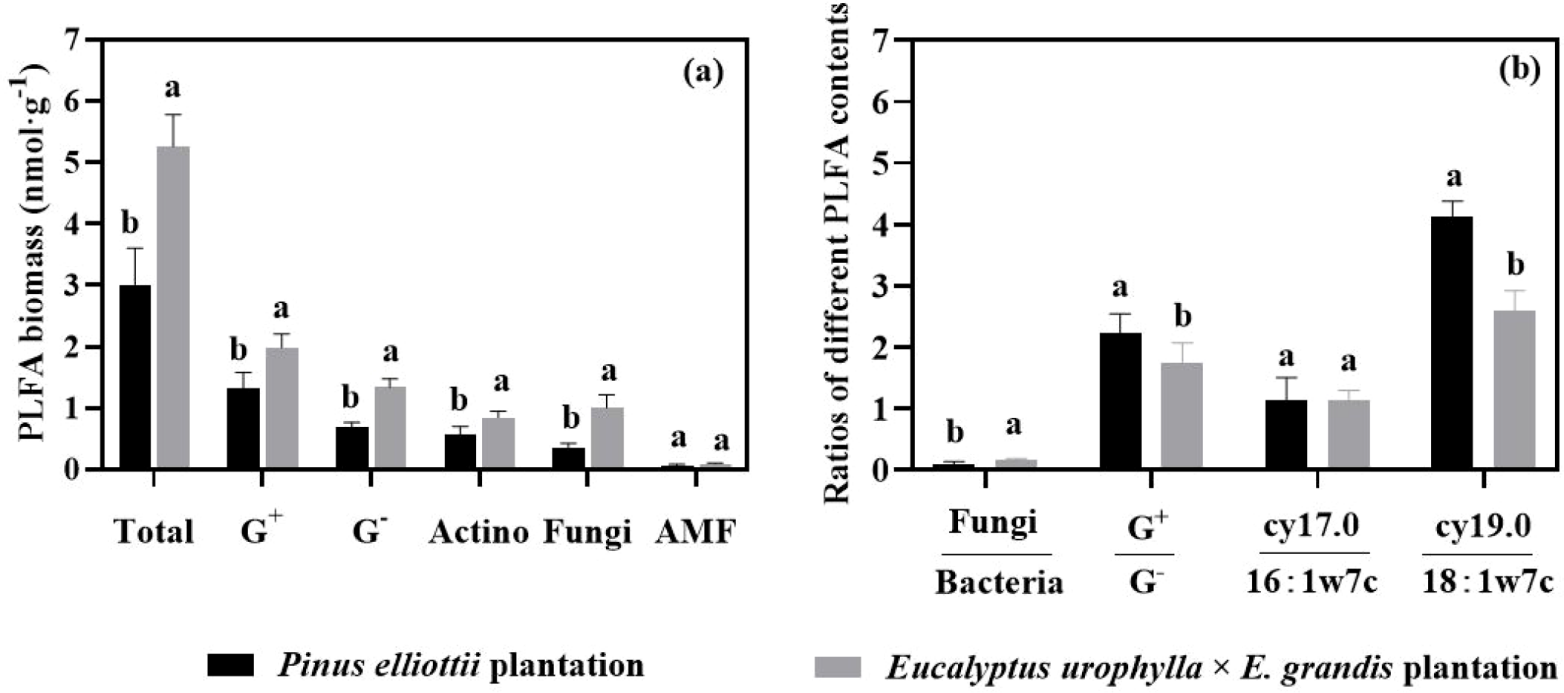
PLFA biomass **(a)** and ratios of different PLFA contents **(b)** in *Pinus elliottii* and *Eucalyptus urophylla × E. grandis* plantations (n=4).

As illustrated in **Figure 7A**, the conversion from *Pinus elliottii* to *Eucalyptus urophylla × E. grandis* plantations significantly increased the total soil phospholipid fatty acid (PLFA) biomass, as well as the biomasses of Gram-positive bacteria, Gram-negative bacteria, actinomycetes, and fungi (all *p* < 0.05). Among these microbial groups, fungi exhibited the most pronounced increase (185.71%), followed by Gram-negative bacteria (95.85%) and total PLFA (74.75%). In contrast, the increase rates of Gram-positive bacteria and actinomycetes were 50% and 47.36%, respectively. Although the biomass of arbuscular mycorrhizal fungi (AMF) increased by 50%, no significant difference was detected between the two stand types (*p >* 0.05).

Tree species conversion induced significant alterations in the ratios of soil microbial PLFAs (**Figure 7B**). Specifically, both the Gram-positive bacteria/Gram-negative bacteria ratio and the cy19:0/18:1w7c ratio were significantly reduced *(p* < 0.05), with the cy19:0/18:1w7c ratio showing the greatest reduction (36.89%). Conversely, the fungi/bacteria ratio was significantly increased by 40% (*p* < 0.05), whereas no significant difference was observed in the cy17:0/16:1w7c ratio between the two stand types (*p >* 0.05). Collectively, these results indicate that the conversion from *Pinus elliottii* to *Eucalyptus urophylla × E. grandis* plantations improved soil fertility, mitigated microbial stress significantly, and enhanced the carbon sink potential of the plantations.

### 3.5 Seasonal variations of soil dissolved organic carbon and soil microbial biomass carbon in two stand types

**Figure 8:**
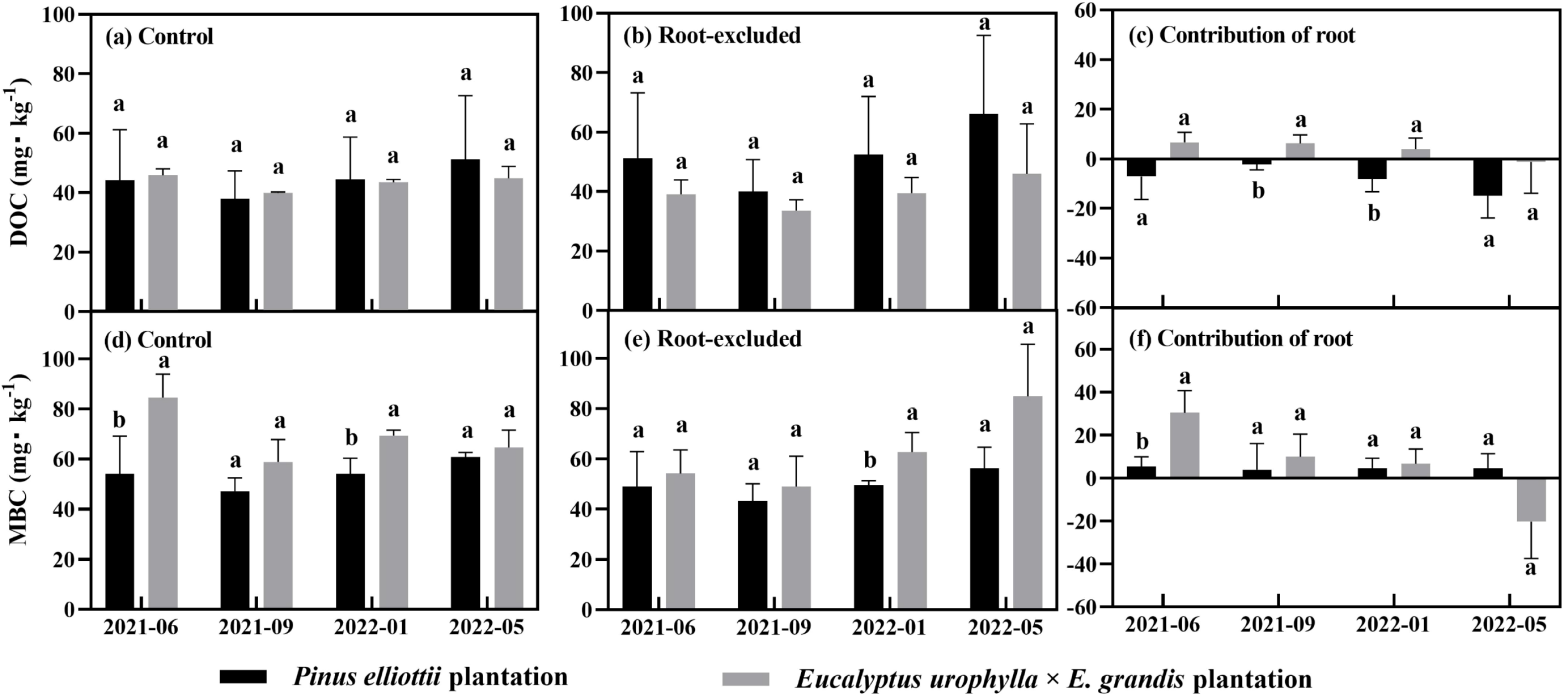
Seasonal variations in DOC **(A–C)** and MBC **(D–F)** in topsoil of *Pinus elliottii* and *Eucalyptus urophylla × E. grandis* plantations (n=4).

Following the conversion from Pinus elliottii to Eucalyptus urophylla × E. grandis plantations, soil dissolved organic carbon (DOC) exhibited distinct seasonal variations, with similar patterns between the two stand types (**Figures 8A–C**). DOC decreased significantly from June to September 2021 and then increased gradually from September 2021 to May 2022, with relatively high values from January to May 2022 and the lowest value in September 2021. Mean DOC content was 68.13 mg·kg^-^¹ in Eucalyptus urophylla × E. grandis, 68.6% higher than the 40.39 mg·kg^-^¹ in Pinus elliottii (p < 0.05). Significant interspecific differences in DOC were observed in June 2021 and May 2022 (p < 0.05). Root-contributed DOC differed significantly between the two species from September 2021 to January 2022 (p < 0.05), and root exclusion reduced DOC by 22.7% in Pinus elliottii and 28.3% in Eucalyptus urophylla × E. grandis.

Seasonal dynamics of soil microbial biomass carbon (MBC) were consistent with DOC, also showing a pattern of decrease first and then increase (**Figures 8D–F**). MBC was relatively high in June 2021 and May 2022, and low from September 2021 to January 2022. Mean MBC in *Eucalyptus urophylla × E. grandis* reached 118.69 mg·kg^-^¹, 181.9% higher than the 42.11 mg·kg^-^¹ in *Pinus elliottii* (*p* < 0.05). Root exclusion significantly reduced MBC in both stands, with a decrease of 31.5% in *Pinus elliottii* and 42.6% in *Eucalyptus urophylla × E. grandis* (*p* < 0.05). The fluctuation amplitude of MBC in *Eucalyptus urophylla × E. grandis* was significantly larger in May 2022, while *Pinus elliottii* showed more stable MBC from September 2021 to January 2022.

### 3.6 Relationships between soil respiration rate and soil temperature and moisture

**Figure 9:**
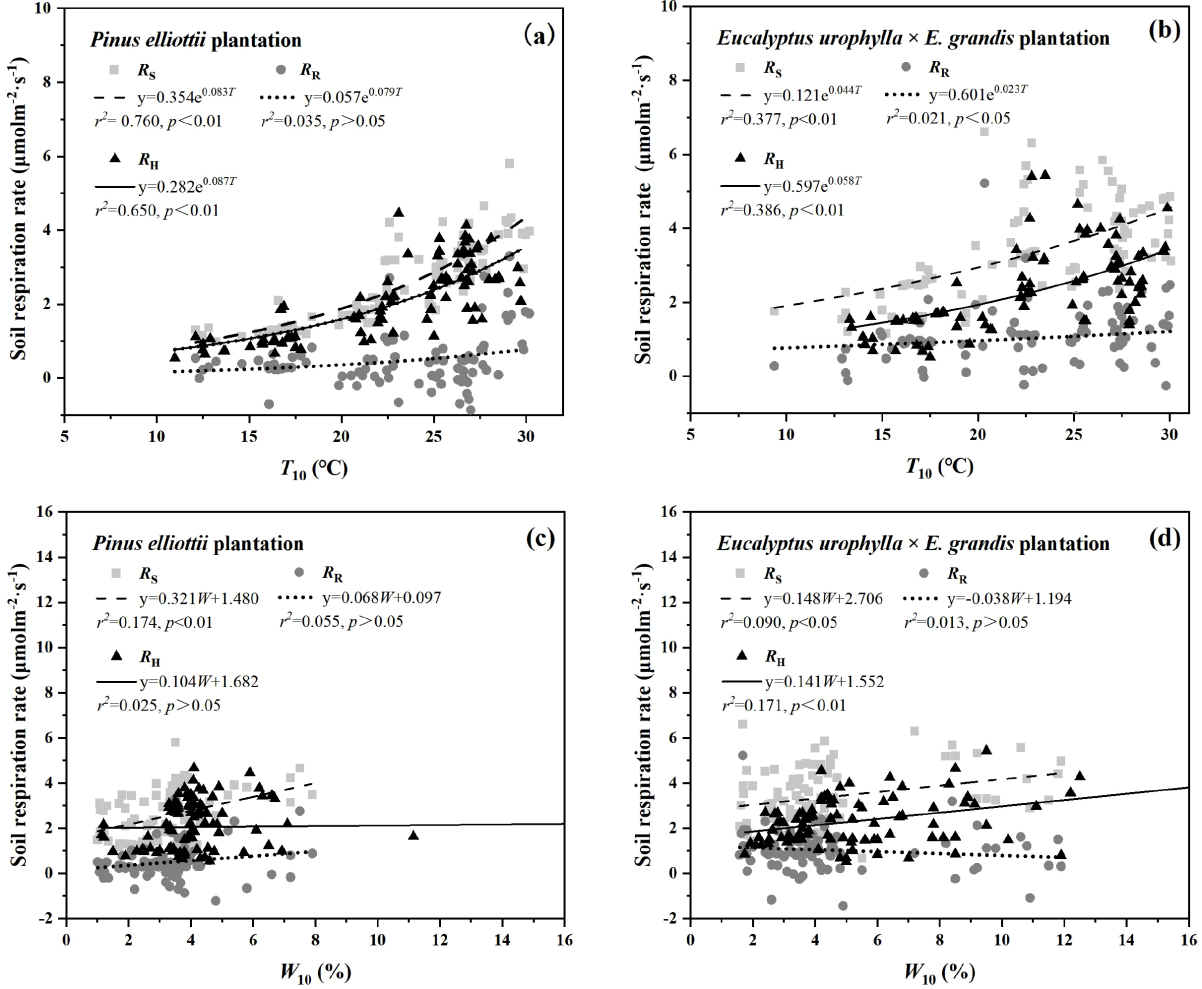
Relationships between soil respiration rate and soil temperature **(a, b)** and moisture **(c, d)** in *Pinus elliottii* and *Eucalyptus urophylla × E. grandis* plantations (n=96).

In both *Pinus elliottii* and *Eucalyptus urophylla × E. grandis* plantations, total soil respiration and its components (total soil respiration and heterotrophic respiration) exhibited significant positive correlations with soil temperature (*T*_10_) (**Figure 9**). Specifically, the fitting effects of total soil respiration (*r*² = 0.760, *p<* 0.01) and heterotrophic respiration (*r*² = 0.650, *p* < 0.01) with *T*_10_ in *Pinus elliottii* plantations were significantly better than those in *Eucalyptus urophylla × E. grandis* plantations (total soil respiration: *r*² = 0.377, *p* < 0.01; heterotrophic respiration: *r*² = 0.386, *p* < 0.01). The effects of soil moisture (*W*_10_) on respiration rate differed between the two stand types: total soil respiration in *Pinus elliottii* plantations was significantly positively correlated with *W*_10_ (*p* < 0.01), and soil moisture explained 17.4% of the variation in respiration. In *Eucalyptus urophylla × E. grandis* plantations, total soil respiration and heterotrophic respiration were positively correlated with *W*_10_ (*p* < 0.05), explaining 9% and 17.1% of the variation in respiration, respectively. In addition, compared with the single-factor models, the bivariate models of soil temperature and moisture significantly improved the explanatory power for the variation of soil respiration components, and all bivariate models reached extremely significant levels except for root respiration in *Pinus elliottii* (*p* < 0.01, **Table 4**).

**Table 4:** Regression models and *Q*_10_ values of soil temperature, moisture and respiration rate (n=96).

Forest type	Component	Exponential model			Linear model			Interactive model				*Q*_10_
		a	B	*r^2^*	a	b	*r^2^*	a	b	c	*r^2^*	
*Pinus elliottii*	Total soil respiration	0.354	0.083	0.760**	0.321	1.480	0.174**	0.338	0.078	0.149	0.785**	2.29
	Root respiration	0.057	0.079	0.035	0.068	0.097	0.055	2.394	0.440	1.680	0.042	2.20
	Heterotrophic respiration	0.282	0.087	0.650**	0.104	1.682	0.025	0.226	0.086	0.171	0.663**	2.38
*Eucalyptus urophylla ×E. grandis*	Total soil respiration	0.121	0.044	0.377**	0.148	2.706	0.090*	1.018	0.043	0.149	0.422**	1.55
	Root respiration	0.601	0.023	0.021*	-0.038	1.194	0.013	0.849	0.026	-0.304	0.050**	1.25
	Heterotrophic respiration	0.597	0.058	0.386**	0.141	1.552	0.171**	0.311	0.059	0.386	0.572**	1.78

In all tables of this study, one asterisk (*) indicates a significant difference at *p* < 0.05, and two asterisks (**) indicate a significant difference at *p* < 0.01.

### 3.7 Relationships between soil respiration rate and environmental factors

**Figure 10:**
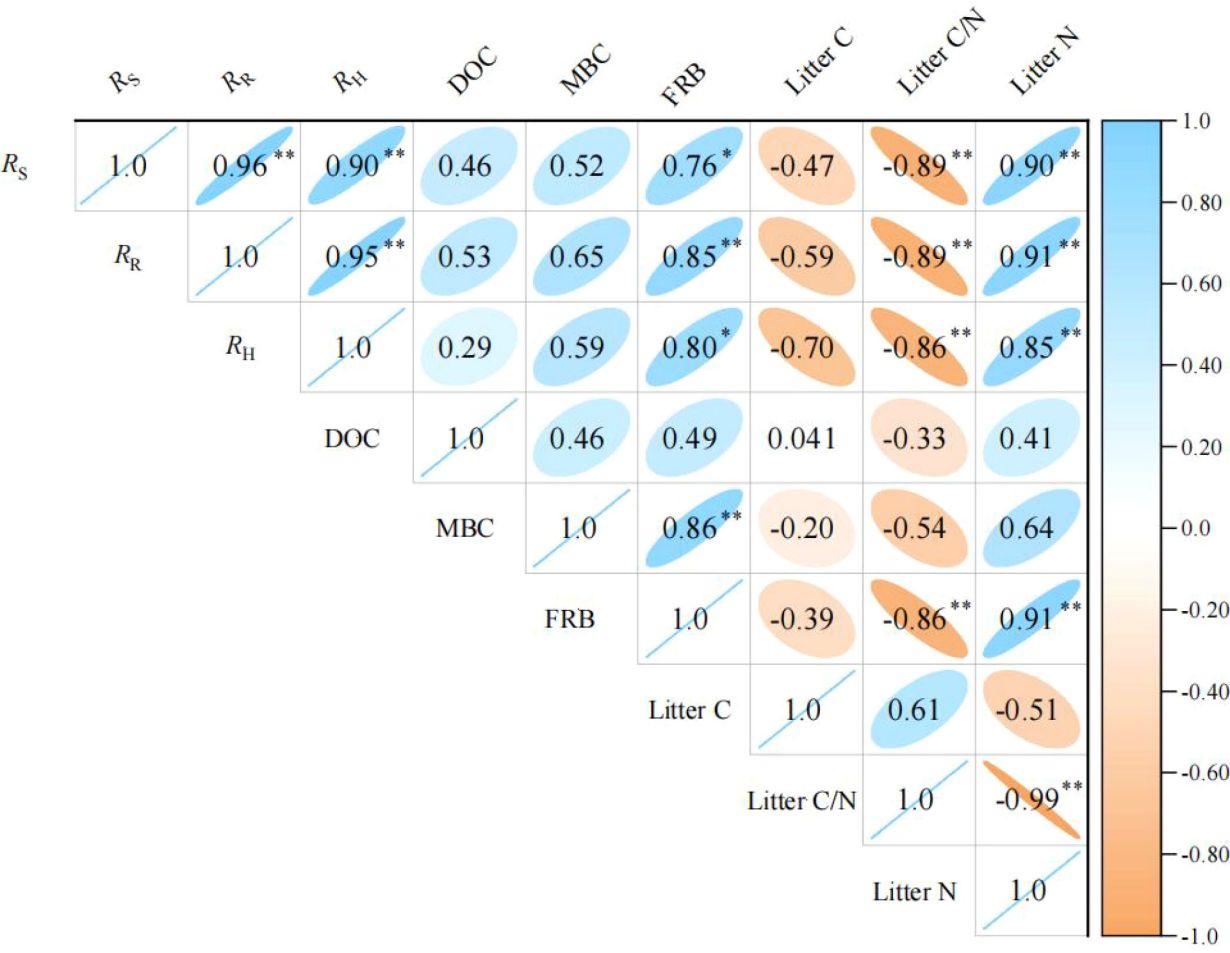
Pearson correlation of soil respiration and environmental factors (n=8). *R*_S_, Total soil respiration rate; *R*_R_, Root respiration rate; *R*_H_, Heterotrophic respiration rate; DOC, Soil dissolved organic carbon content; MBC, Soil microbial biomass carbon content; FRB, Fine root biomass; Litter C, Litter carbon content; Litter N, Litter nitrogen content; Litter C/N, Litter carbon-to-nitrogen ratio. Fine root biomass refers to the biomass of live small roots with a diameter < 2 mm, measured in the 0–10 cm soil layer in 2021. MBC and DOC represent the average values of soil microbial biomass carbon and soil dissolved organic carbon, respectively, measured in the 0–10 cm soil layer during the four quarters from 2021 to 2022. * indicates a significant difference at *p* < 0.05, and ** indicates a significant difference at *p* < 0.01.

As illustrated in **Figure 10**, the total soil respiration rate (*R*_S_) was extremely significantly and positively correlated with root respiration rate (*R*_R_) and heterotrophic respiration rate (*R*_H_) in both stand types (correlation coefficient *r* = 0.96 and 0.90, respectively, *p* < 0.01). Additionally, an extremely significant positive correlation was also observed between *R*_R_ and *R*_H_ (*r* = 0.95, *p* = 0.001), indicating that all components of soil respiration are highly synergistic in regulating the process of soil carbon release. Fine root biomass (FRB) was significantly and positively correlated with *R*_S_, *R*_R_, and *R*_H_ (all *p* < 0.05), and was also extremely significantly and positively correlated with soil microbial biomass carbon (MBC) (*r* = 0.86,*p* = 0.006). This suggests that fine roots can enhance soil respiration levels through a dual pathway: directly regulating root respiration and indirectly promoting microbial metabolism. The correlation between litter carbon content (Litter C) and all components of soil respiration was not significant (*p* > 0.05), whereas litter carbon-to-nitrogen ratio (Litter C/N) was extremely significantly and negatively correlated with *R*_S_, *R*_R_, and *R*_H_ (*p* < 0.01), and litter nitrogen content (Litter N) was extremely significantly and positively correlated with them (*p* < 0.01). These results reflect that litter quality is a key factor regulating soil respiration. Furthermore, the correlation between soil dissolved organic carbon (DOC) and MBC was not significant (*r* = 0.46, *p* = 0.252), suggesting that their coupling relationship may be regulated by other environmental factors.

**Figure 11:**
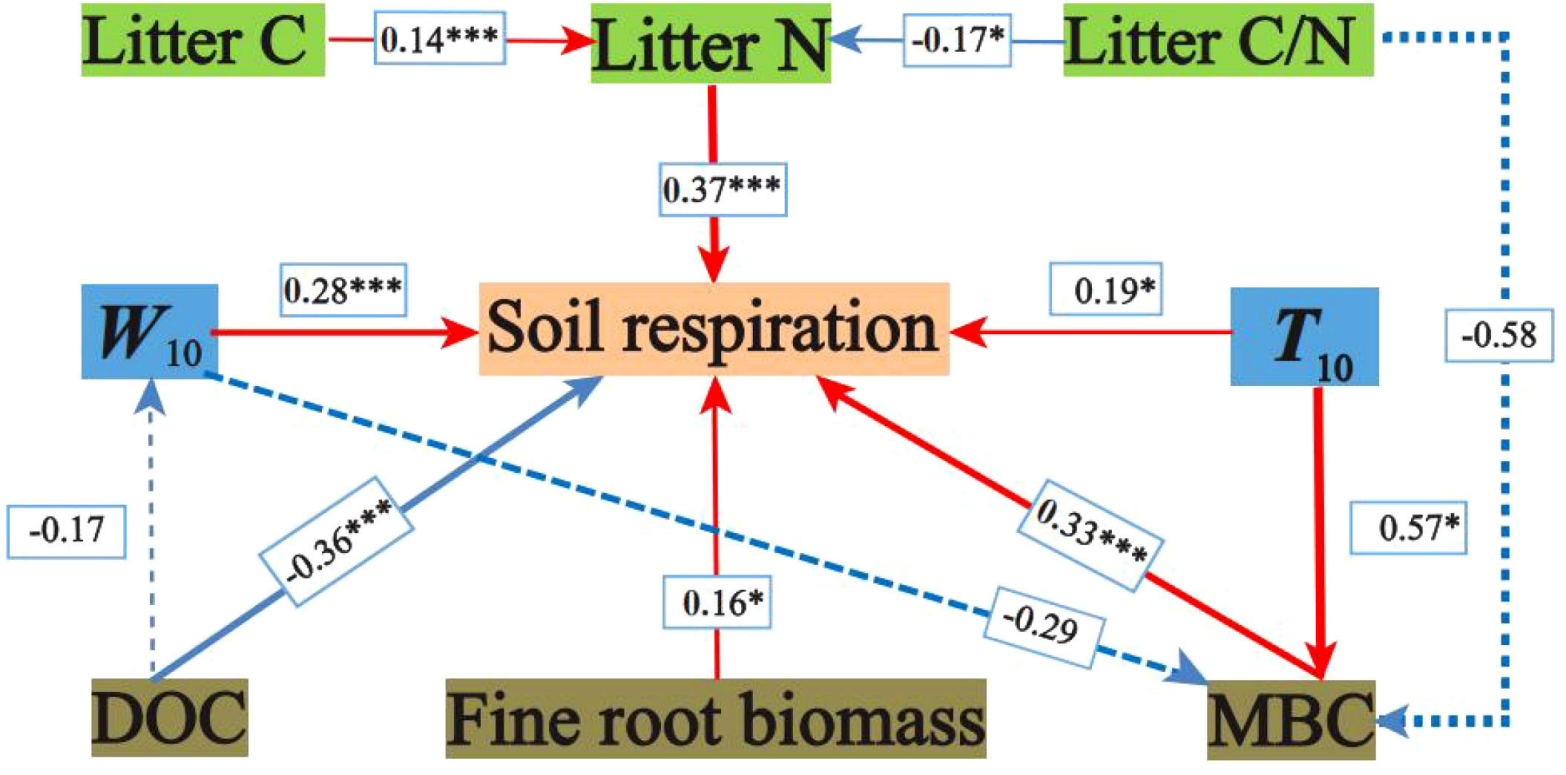
Structural equation model of soil respiration and environmental factors (n=16). The SEM, structural equation model was used to clarify the causal relationships between soil respiration and related environmental factors, including litter quality, fine root biomass, soil organic carbon pool, *T*_10_, soil temperature, and *W*_10_, soil moisture. In the SEM diagram (**Figure 11**), red arrows represent positive correlation pathways, blue arrows represent negative correlation pathways, solid arrows denote significant pathways (*p* < 0.05), and dashed arrows represent non-significant pathways (*p*> 0.05). The numbers marked on the arrows are standardized path coefficients. The model fitting parameters are as follows: *χ*² = 14.145, *df*, degrees of freedom = 11; CFI, comparative fit index = 0.973; TLI, Tucker-Lewis index = 0.926; AIC, Akaike information criterion = 138.8; BIC, Bayesian information criterion = 157.3. The significance levels are indicated as follows: *p < 0.05, **p < 0.01, ***p < 0.001.

We employed a structural equation model (SEM) to systematically analyze the regulatory mechanisms between soil respiration and environmental factors, including litter quality, fine root biomass, soil organic carbon pool, soil temperature, and soil moisture (**Figure 11**). The model fitting results (*χ*² = 14.145, *df* = 11, CFI = 0.973, TLI = 0.926) indicated that the model fitted well with the observed data and could effectively characterize the causal relationships among variables. From the perspective of direct effects, litter nitrogen (Litter N, *b* = 0.37, *p* < 0.05), soil microbial biomass carbon (MBC, *b* = 0.33, *p* < 0.05), and soil moisture (*W*_10_, *b* = 0.28, *p* < 0.05) were the dominant positive driving factors for soil respiration. Soil temperature (*T*_10_, *b* = 0.19, *p* < 0.05) and fine root biomass (*b* = 0.16, *p* < 0.05) exerted relatively weak positive effects. Notably, dissolved organic carbon (DOC) showed a significant negative direct effect on soil respiration (*b* = −0.36, *p* < 0.05), which was mainly attributed to the consumption of labile DOC for microbial growth and enzyme synthesis rather than direct stimulation of respiration. In the coastal sandy soil with low nutrient availability, DOC was preferentially used as a carbon and energy source for microbial metabolism, thereby reducing its direct contribution to CO_2_ release.

From the perspective of indirect regulatory pathways, soil temperature promoted soil respiration mainly through the positive cascading pathway of *T*_10_ → MBC → respiration (*b* = 0.57, *p* < 0.05). Litter quality (Litter C/N) indirectly regulated respiration by controlling substrate availability and litter N content. According to the total standardized effects (direct + indirect), the **contribution magnitude of key factors to soil respiration followed the order**: MBC > Litter N > DOC > soil moisture > fine root biomass > soil temperature. These results confirm that soil respiration was co-regulated by multiple biological and environmental factors, with microbial and substrate-related factors being the dominant drivers.

## 4 Discussion

### 4.1 Tree species functional type conversion synergistically drives accelerated soil carbon cycling through improved aboveground litter quality and surge in belowground fine root input

In a subtropical coastal sandy area with consistent site conditions, soil texture, and management history, this study found that the conversion from Pinus elliottii (coniferous tree) to Eucalyptus urophylla × E. grandis (broadleaved tree) significantly increased the annual total soil respiration flux by 25.52%. This increase was mainly attributed to the sharp rise in root respiration (an increase of 62.74%), while heterotrophic respiration only increased slightly (9.01%). Notably, although the observation in this study that “coniferous-to-broadleaved forest conversion leads to enhanced soil respiration” is consistent with previous research results (Raich and Tufekciogul, 2000; Huang et al., 2014; Yu et al., 2014), the underlying mechanisms differ slightly. Previous studies generally attributed the enhanced respiration to the dual driving forces of improved aboveground litter quality and increased belowground root activity (Sheng et al., 2010; Huang et al., 2014; Liu et al., 2019), and this overall framework is highly consistent with the present study. However, this study further clarifies a subtle mechanistic difference: the substantial increase in belowground fine root biomass acts as the dominant driver, with a much greater contribution than aboveground litter, whereas earlier studies did not distinguish such clear primary and secondary roles. The aboveground litter of Eucalyptus urophylla × E. grandis plantations had increased nitrogen content and decreased lignin/C:N ratio (**Table 1**), which significantly improved substrate decomposability; meanwhile, the substantial increase in belowground fine root biomass directly enhanced root respiration and induced microbial activity (Li et al., 2025).

Quantitative analysis further clarified the relative contributions and interactive relationships of these coupled drivers. Fine root biomass alone explained 73.9% (*r*² = 0.739) of the variation in total soil respiration, and was extremely significantly and positively correlated with root respiration (*r* = 0.92, *p* < 0.01) and heterotrophic respiration (*r* = 0.85, *p* < 0.01), demonstrating its dominant role in regulating soil respiration. Litter C/N ratio was significantly negatively correlated with total soil respiration (*r* = −0.76, *p* < 0.01) and microbial biomass carbon (MBC, *r* = −0.71, *p* < 0.01), indicating that litter quality controlled approximately 51.8% of the variation in microbial activity by regulating substrate availability.

The surge in belowground fine root biomass was the primary factor driving the enhancement of respiration. The fine root biomass of *Eucalyptus urophylla × E. grandis* (2.98 t·hm^-^²) was 272.5% higher than that of *Pinus elliottii* (0.80 t·hm^-^²), and it was extremely significantly and positively correlated with total soil respiration, root respiration, and heterotrophic respiration (*r*² = 0.86, *p* = 0.006), indicating its key regulatory role in this system. Fine roots accelerated carbon turnover through dual pathways: first, their own metabolic activities directly resulted in the root respiration rate of *Eucalyptus urophylla × E. grandis* (average 1.01 μmol·m^-^²·s^-^¹) reaching 2.5 times that of *Pinus elliottii* (0.39 μmol·m^-^²·s^-^¹); second, the process of fine root turnover released a large number of root exudates and residues, inducing the rhizosphere priming effect and significantly activating the microbial community (Billings et al., 2024). This is supported by the measured data: the soil dissolved organic carbon (DOC = 68.13 mg·kg^-^¹) and microbial biomass carbon (MBC = 118.69 mg·kg^-^¹) in *Eucalyptus urophylla × E. grandis* plantations were significantly higher than those in *Pinus elliottii* plantations (DOC = 40.39 mg·kg^-^¹; MBC = 42.11 mg·kg^-^¹), and MBC was significantly positively correlated with fine root biomass, thereby verifying the underground driving chain of “increased fine root input → improved microbial activity and biomass → enhanced heterotrophic respiration”.

The systematic improvement of aboveground litter quality provided high-activity substrates. Although there was no significant difference in annual litter production between the two stand types (*p* = 0.60), their chemical properties underwent fundamental changes. The litter nitrogen content of *Eucalyptus urophylla × E. grandis* (9.20 g·kg^-^¹) was 39.4% higher than that of *Pinus elliottii*, the carbon-to-nitrogen ratio (51.55) and lignin/nitrogen ratio (19.55) were reduced to 66.8% and 35.3% of those of *Pinus elliottii*, respectively, and the lignin content (17.99%) was only 49.2% of that of *Pinus elliottii* (36.59%). This chemical property of low lignin and low C/N greatly improved the decomposability of litter and provided a more easily utilizable carbon source for microorganisms (Börger et al., 2022), thereby contributing to heterotrophic respiration.

The aboveground and belowground factors did not act independently, but interacted synergistically. Improved litter quality optimized the soil microenvironment and enhanced microbial responsiveness, which amplified the rhizosphere priming effect induced by fine roots. Meanwhile, increased fine root input further promoted microbial growth and activity, strengthening the decomposition of high-quality litter. Structural equation modeling confirmed that fine root biomass and litter quality together regulated soil respiration through direct and indirect pathways, with a total explanatory power of 68.2% for respiratory variation. This study indicated that, in the nutrient-limited coastal sandy environment, tree species conversion simultaneously optimizes aboveground litter quality and surges underground fine root biomass. The combination of aboveground high-activity litter input and underground high-intensity fine root carbon input and priming effect jointly promotes the rapid microbial turnover of soil carbon, ultimately forming a carbon cycle acceleration model of “high-activity carbon input – rapid microbial turnover – strong respiratory output”.

### 4.2 Nonlinear temperature response of microbial carbon use efficiency is the underlying mechanism regulating the temperature sensitivity of heterotrophic respiration

Although the soil respiration intensity of *Eucalyptus urophylla × E. grandis* plantations was significantly higher than that of *Pinus elliottii* plantations, its temperature sensitivity (*Q*_10_) decreased comprehensively: the *Q*_10_ of total respiration decreased from 2.29 to 1.55, the *Q*_10_ of root respiration decreased from 2.20 to 1.25 (nearly temperature-insensitive), and the *Q*_10_ of heterotrophic respiration also decreased from 2.38 to 1.78. This coexistence phenomenon of “high respiration intensity and low temperature sensitivity” cannot be fully explained by the traditional “carbon quality-temperature” hypothesis (Li et al., 2017; Brangarí and Rousk, 2025). This study introduced the microbial carbon use efficiency (CUE) theory and attempted to reveal its potential mechanism from the perspective of microbial metabolic allocation. CUE refers to the proportion of carbon absorbed by microorganisms that is converted into their own biomass, and the rest is released as CO_2_ through respiration. Recent studies have shown that the response of CUE to temperature is not monotonically increasing, but there is a critical threshold around 15 °C: above this threshold, microorganisms can achieve thermal adaptation by adjusting enzyme systems and membrane lipid composition, CUE is significantly improved, and biomass synthesis is accelerated; below this threshold, CUE is relatively insensitive to temperature changes (Lee et al., 2023; Pei et al., 2025). The average annual temperature in the study area is 21.9 °C, and the 10 cm soil temperature reaches 25.0–29.9 °C in summer, which is much higher than the threshold, while it is 13.0–15.5 °C in winter, which is at the edge of the threshold — this temperature pattern provides an ideal habitat for testing the nonlinear response of CUE.

Combining substrate conditions, microbial community indicators, and theoretical expectations, the following mechanistic inferences can be formed. In *Pinus elliottii* plantations, litter has high lignin content (36.59%), high C/N ratio (77.12), low fine root biomass (0.80 t·hm^-^²), and insufficient available carbon sources for microorganisms, resulting in a weak correlation between microbial biomass carbon and fine root biomass (*r*² = 0.49). Under this substrate limitation, even at high summer temperatures, the potential for CUE improvement is limited, and heterotrophic respiration mainly depends on the enhancement of extracellular enzyme activity driven by temperature, thus showing a high *Q*_10_ (2.20) (Li et al., 2021). In contrast, *Eucalyptus urophylla × E. grandis* plantations provide sufficient and easily utilizable carbon sources for microorganisms by virtue of high-quality litter (low lignin, low C/N) and high fine root input (2.98 t·hm^-^²). Measurements showed that the total soil PLFA of this stand increased from 3.01 to 5.26 nmol·g^-^¹, the fungal abundance increased by 185.71%, the microbial biomass carbon (MBC) increased significantly, and it was strongly positively correlated with fine root biomass (*r*² = 0.86). These changes are highly consistent with the theoretical expectation of accelerated microbial biomass accumulation under improved CUE conditions (Ren et al., 2024). Based on this, it is inferred that in the high-temperature season, the microorganisms in *Eucalyptus urophylla × E. grandis* plantations break through substrate limitation, achieve a leap in CUE, and allocate more carbon to biomass synthesis rather than respiratory release; therefore, although the absolute intensity of heterotrophic respiration increases, its dependence on temperature decreases (*Q*_10_ = 1.78). The structural equation model further supports the above inference: soil temperature mainly affects respiration rate indirectly through microbial pathways, indicating that microbial processes play a core mediating role in regulating the temperature sensitivity of respiration. It should be noted that the decrease in *Q*_10_ may also be related to changes in microbial community composition, the relief of substrate limitation at high temperatures, or changes in the proportion of respiratory components, and there may be superposition or interaction between different mechanisms. Therefore, this study proposes the nonlinear temperature response of CUE as the most explanatory mechanism hypothesis under the current data conditions, which still needs to be verified in the future through methods such as isotope tracer culture, direct CUE determination, or microbial physiological trait analysis.

In summary, the nonlinear temperature response of CUE provides an integrated perspective with both theoretical depth and empirical support for explaining the coexistence phenomenon of “high respiration intensity and low temperature sensitivity”, and also provides a new cognitive framework for predicting soil carbon dynamics in plantations under the background of climate change.

### 4.3 The special habitat of coastal sandy land enhances the regulatory effect of tree species conversion on microbial-carbon cycling

Compared with inland forests, coastal sandy lands have typical fragile characteristics such as poor soil, salt stress, weak water-holding capacity (volumetric water content only 1.01%–11.8%), and strong thermal conductivity, which significantly amplify the limiting effect of biological factors on carbon cycling. This study found that *Eucalyptus urophylla × E. grandis* plantations significantly increased soil pH, organic carbon and total nitrogen contents, alleviating the acidification and nutrient limitation accumulated in *Pinus elliottii* plantations for a long time; at the same time, soil moisture was also significantly increased (*p* = 0.01). Although the background constraint of weak water-holding capacity in sandy lands still exists, this relative improvement still provides more favorable water conditions for microbial thermal adaptation. At the same time, sandy soil has fast thermal conductivity, and the surface soil temperature in summer is 5–8 °C higher than the air temperature, making microorganisms in a state of “strong thermal adaptation” for a long time, and the leap range of CUE may be higher than that in clay or loam ecosystems. In this context, even in the face of seasonal drought stress, microorganisms can maintain basic metabolism by adjusting cell membrane permeability, ensuring that the regulation of CUE on heterotrophic respiration remains effective.

More importantly, due to the extremely small initial nutrient pool of coastal sandy lands, the “marginal effect” of the quality and quantity of litter and fine roots on microbial activity is much higher than that of fertile soil (Gao et al., 2020),.No universal quantitative thresholds for microbial critical activation are available in the literature, as thresholds vary substantially with soil texture, organic carbon content, and nutrient status (Fontaine, 2003; Blagodatskaya and Kuzyakov, 2008). However, our observations clearly indicate that critical activation has been triggered: fine root biomass increased by 272.5%, fungal PLFA increased by 185.71%, microbial biomass carbon increased nearly threefold, and litter C/N and lignin content decreased to levels favorable for rapid decomposition (Blagodatskaya and Kuzyakov, 2008; Wang et al., 2015; Qin et al., 2024). *Eucalyptus urophylla × E. grandis* generated such a pronounced respiratory response within a short period merely by improving these two key carbon input pathways (Bai et al., 2024). Although the relative increase in heterotrophic respiration (9.01%) was lower than that of root respiration (62.74%) in this study, the increase in its absolute value remains ecologically significant — on nutrient-poor sandy substrates, even a small increase in available carbon input can easily trigger the “critical activation” of the entire microbial community (Gao et al., 2020; Ren et al., 2024; Brangarí et al., 2025). Therefore, the environmental stress and resource limitation characteristic of coastal sandy lands do not weaken the effects of tree species conversion; instead, they amplify the regulatory capacity of functional tree species on belowground ecological processes, further highlighting the critical importance of scientific tree species selection in fragile ecosystems.

### 4.4 Tree species conversion can achieve the unity of high carbon turnover and high carbon sequestration stability, supporting the multifunctional optimization of coastal protection forests

Ecological restoration often faces the trade-off dilemma between “improving productivity” and “enhancing carbon sink stability”. This study provides a possible synergistic path: although the conversion from *Pinus elliottii* to *Eucalyptus urophylla × E. grandis* significantly increased soil respiration (carbon output), due to the significant decrease in the *Q*_10_ of heterotrophic respiration and total respiration, under the future climate warming scenario, the soil carbon emission of this stand will respond more gently to warming, and the carbon release risk will be relatively lower. At the same time, the rapidly improved microbial activity and nutrient cycling efficiency help to improve the structure and fertility of sandy soil in a short period of time, enhance the wind erosion resistance and salt tolerance of the stand, and thus improve the protective function. Thus, *Eucalyptus urophylla × E. grandis* plantations initially present a positive regulatory pattern of “high-activity carbon input (high-quality litter + high-quantity fine roots) → rapid microbial turnover (high MBC, high PLFA) → low carbon warming response (low *Q*_10_)”, providing a potential path for balancing ecological protection and climate mitigation.

Based on the above findings, this study puts forward two management suggestions for the existing large-area pure *Pinus elliottii* plantations: first, carry out mixed transformation by introducing broadleaved tree species, or implement litter layer regulation (such as retaining broadleaved litter cover) to directionally optimize underground carbon processes; Second, our results highlight the need for carbon cycle models to explicitly separate root respiration and heterotrophic respiration in climate-vulnerable ecosystems such as coastal sandy lands. This is a methodological recommendation for future modeling studies, rather than a forest management measure, to reduce bias in predicting soil carbon dynamics. In summary, this study not only verifies the universal law of tree species functional type regulating soil respiration, but also reveals the unique mechanism of “fine root-microbe-CUE” coupling mediation in the special habitat of coastal sandy lands, providing a scientific basis for the improvement of carbon sink function and adaptive management of coastal protection forests under the background of global change.

### 4.5 Limitations of this study

Several limitations should be acknowledged in this study. First, the paired adjacent plot design, while ensuring comparability in stand age and soil texture, may still introduce uncontrolled confounding variables (e.g., historical microenvironmental heterogeneity). Although such effects are expected to be minor given the uniform soil type and stand history in this study, they may still slightly reduce the robustness of statistical comparisons. Future studies could adopt randomized complete block design or increase replication to further minimize such bias. Second, the scope of considered environmental factors was relatively limited; we did not comprehensively incorporate some key regulators in coastal sandy lands, such as soil salinity dynamics, nutrient availability, and complex interspecific biological interactions. These factors may mediate soil carbon processes but are unlikely to alter the core conclusion that fine root biomass and litter quality dominate soil respiration after conversion. Subsequent research can integrate long-term salinity monitoring and nutrient manipulation experiments to strengthen mechanistic explanations. Third, although we proposed a mechanistic framework involving microbial carbon use efficiency (CUE) in regulating soil respiration and its temperature sensitivity, CUE was not directly measured. This missing direct evidence brings moderate uncertainty to the interpretation of thermal adaptation mechanisms, but our inference is well supported by observed changes in microbial biomass, community structure, and *Q*_10_. Future measurements using isotopic tracing or direct CUE determination would help validate this mechanism. Finally, our conclusions are site-specific to subtropical coastal sandy lands. This limits the direct extrapolation to other coastal or climatic regions, but our findings remain highly representative for similar subtropical sandy protection forests. To improve broader applicability, multi-site comparisons across coastal zones are recommended in future research.

## 5 Conclusions

This study clarified the effects and driving mechanisms of tree species conversion from *Pinus elliottii* to *Eucalyptus urophylla × E. grandis* on soil carbon cycling in subtropical coastal sandy lands. The results showed that this conversion significantly increased annual total soil respiration flux by 25.52%, along with significant increases in root respiration (+62.74%) and heterotrophic respiration (+9.01%). The increase was dominated by root respiration, which was driven by the 272.5% increase in fine root biomass, coupled with improved litter quality (lower C/N ratio and lignin content) and enhanced soil microbial activity.

Notably, tree species conversion significantly reduced the temperature sensitivity of soil respiration: the *Q*_10_ of total soil respiration decreased from 2.29 to 1.55, and root respiration became nearly temperature-insensitive with a *Q*_10_ of only 1.25, thereby markedly improving the stability of ecosystem carbon sequestration. Meanwhile, the explanatory power of soil temperature for respiration variation decreased significantly from 76% to 37.7%, whereas the regulatory roles of litter quality, fine root biomass, and microbial activity became dominant. Soil moisture was not a limiting factor throughout the study period.

Quantitatively, fine root biomass alone explained 73.9% of the variation in total soil respiration, and litter C/N ratio was significantly negatively correlated with microbial biomass carbon (*r* = −0.71, *p* < 0.01). Structural equation modeling confirmed that the combined effects of litter quality, fine root biomass, and microbial activity had a total explanatory power of 68.2% for soil respiration variation.

This conversion achieves a virtuous cycle of “high carbon turnover and high carbon sequestration stability” by enhancing soil respiration while reducing its temperature dependence. These findings provide key scientific support for the optimal configuration of coastal protection forests, soil improvement, and carbon sequestration enhancement in fragile coastal sandy ecosystems. Further research is recommended to focus on the seasonal dynamics of fine roots and long-term monitoring of microbial community evolution to deepen the mechanistic understanding.

## Data Availability

The original contributions presented in the study are included in the article/**Supplementary Material**. Further inquiries can be directed to the corresponding author/s.

## Glossary

C: Carbon
N: Nitrogen
FRB: Fine Root Biomass
DOC: Dissolved Organic Carbon
MBC: Microbial Biomass Carbon
PLFA: Phospholipid Fatty Acid
AMF: Arbuscular Mycorrhizal Fungi
G⁺: Gram-positive Bacteria
G⁻: Gram-negative Bacteria
Actino: Actinobacteria
*Q*₁₀: Temperature Sensitivity of Soil Respiration
*R_S_*: Total Soil Respiration Rate
*R_R_*: Root Respiration Rate
*R_H_*: Heterotrophic Respiration Rate
SEM: Structural Equation Model
ANOVA: Analysis of Variance
CFI: Comparative Fit Index
TLI: Tucker-Lewis Index
AIC: Akaike Information Criterion
BIC: Bayesian Information Criterion
TDR: Time-Domain Reflectometer.
